# Mechanisms of Gas-Induced Posterior Vitreous Detachment: A Look Behind the Bubble Using Optical Coherence Tomography in Prone Position

**DOI:** 10.3390/jcm15041350

**Published:** 2026-02-09

**Authors:** Julian Elias Klaas, Jakob Siedlecki, Benedikt Schworm, Nikolaus Feucht, Mathias Maier, Siegfried G. Priglinger

**Affiliations:** 1 Department of Ophthalmology, LMU University Hospital, Ludwig-Maximilians-University, 80539 Munich, Germany; jakob.siedlecki@med.uni-muenchen.de (J.S.); benedikt.schworm@med.uni-muenchen.de (B.S.); siegfried.priglinger@med.uni-muenchen.de (S.G.P.); 2 Department of Ophthalmology, Technical University, 80333 Munich, Germany; nikolaus.feucht@gmail.com (N.F.); mathias.maier@mri.tum.de (M.M.)

**Keywords:** expansile gas, face-down imaging, full thickness macular hole, FTMH, macular hole, OCT, pneumatic vitreolysis, prone position, posterior vitreous detachment, release, SD-OCT, vitreomacular traction, VMT

## Abstract

**Objectives:** We aimed to visualize the interaction of intravitreal gas with the adjacent vitreomacular interface by using prone position (PP) SD-OCT and suggest possible mechanisms of action behind gas-induced posterior vitreous detachment (PVD) in pneumatic vitreolysis (PV). **Methods:** This was a descriptive–interpretative morphological study. Spectral domain OCT imaging in PP was carried out using a flexible scanning module (SD-OCT-Flex, Heidelberg Engineering) originally designed for bedside imaging. Routine imaging in sitting position was carried out using a regular SD-OCT-device (Heidelberg Engineering). Patients with symptomatic vitreomacular traction (VMT) scheduled for PV with perfluoropropane (C3F8, 0.3 mL) received both sitting and PP imaging immediately before and at regular follow-up visits during the first 3 post-procedural weeks, beginning 3 h after PV. Imaging was reviewed for positional changes of the gas bubble, posterior hyaloid membrane (PHM), VMT configuration, and retrohyaloidal fluid (RHF). **Results:** Three consecutive patients with VMT were included (age: 79, 80, 82 years). Before the procedure, no positional alterations were detected. After the intravitreal injection of gas, PP allowed for the precise discrimination of the PHM and the posterior border of the gas bubble. In contrast to regular SD-OCT in sitting position, PP imaging showed a flattened VMT by the gas bubble with consecutive displacement of RHF from the macular region to the midperiphery. **Conclusions:** This exploratory study describes PP imaging as a tool for the assessment of the morphologic dynamics between the posterior hyaloid membrane, retina, and gas bubble in pneumatic vitreolysis. PP in pneumatic vitreolysis causes the gas bubble to flatten the VMT and to push retrohyaloidal fluid to the midperiphery, possibly allowing for the release of persistent vitreoretinal adhesions and consequent PVD induction.

## 1. Introduction

The process of age-dependent posterior vitreous detachment (PVD), generally defined as the separation of the posterior hyaloid membrane (PHM) from the internal limiting membrane (ILM) of the retina, plays a critical role in multiple pathophysiologic pathways leading to a broad spectrum of vitreoretinal diseases [1,2,3,4,5,6,7]. Over the past few years, our biochemical and clinical understanding of PVD has constantly evolved due to the rapid improvement in high-resolution imaging technologies such as spectral domain and swept-source OCT. For example, Tsukahara et al. used novel widefield OCT and montaging techniques to localize the first morphological signs of PVD at a much younger age and, most importantly, in the paramacular to peripheral regions rather than in the perifoveal regions as previously thought [2,8]. The recent immunohistochemical identification of the posterior hyaloid face as a true basement membrane has further contributed to the way we discuss PVD and its associated diseases [9].

Clinically, traction-related vitreomacular disease has long been treated with induction of PVD by complete surgical removement of the vitreous. The approval of Ocriplasmin by the FDA and EMA in 2012 and 2013, and its recent discontinuation in the USA and Europe in 2020 and 2023, respectively, has sparked interest in alternative methods of PVD induction [10,11]. One such method, intravitreal injection of expansile gas, was first described in 1995 by Chan et al. as an effective treatment for eyes with impending or full thickness macular hole (FTMH) [12,13]. This pilot study documented successful PVD induction in 18 out of 19 patients without previous PVD (in between 2 and 9 weeks) and named the procedure accordingly “pneumatic detachment of the vitreous” [12]. As an inexpensive, readily available, and easily performed procedure, pneumatic vitreolysis (PV)—as it is currently called—has shown respectable PVD rates in a number of retrospective studies [12,14]. On the other hand, a recent randomized clinical trial found a questionable safety profile with retinal detachment rates of about 12% [15]. While its role in vitreomacular disease has yet to be demonstrated, its potential in prophylactic PVD induction in the context of different vitreoretinal disorders makes it essential for both clinicians and scientists to understand the mechanisms by which the intravitreal bubble of gas would release vitreomacular traction and ultimately lead to complete posterior vitreous detachment.

The aim of this morphological study was to visualize the dynamic interaction of intravitreal gas with the vitreomacular interface in prone position (PP) and to deduce possible mechanisms of action behind pneumatically induced PVD.

## 2. Materials and Methods

This retrospective imaging study reports the morphological results of PP Imaging with SD-OCT on the basis of three consecutive patients with symptomatic VMT who opted and were scheduled for PV with 0.3 mL of 12% C3F8. This study adhered to the Declaration of Helsinki. Approval of the institution’s ethics board and informed consent from each patient was obtained.

### 2.1. Subjects

Eligible patients reported continuous subjective impairment of visual function, including visual acuity and metamorphopsia. They were offered primary observation with 3 months of follow-up, the injection of expansile gas, or pars plana vitrectomy. Two patients exhibited persistent (bilateral) VMT for more than three months (case a and case b). One patient opted for primary treatment with PV, despite very limited tractive macular disease and concomitant advanced atrophic AMD (case c). Intravitreal injection of 0.3 mL of expansile gas was conducted in the operating room, in accordance with our clinical hygiene standards. The patients were positioned in the supine position with their eyes oriented nasally upward and received limbal paracentesis immediately following the intravitreal injection. All patients were instructed to maintain a head-tilted-down position for a minimum of 45 min after surgery and at least three times a day as long as a gas bubble was perceived [6].

### 2.2. OCT in Prone Position

OCT images were obtained in the prone position by an experienced clinician and photographer through a dilated pupil, using a flexible SD-OCT module (Spectralis SD-OCT, Flex-Modul, Heidelberg Engineering^®^, Heidelberg, Germany). Since this module was originally designed for bedside imaging in the supine position, there is currently no medical device capable of holding the patient’s head in a horizontally tilted-down position. Consequently, modifications were made to medical walking frame equipment in order to obtain optimal scan quality while minimizing head movements and physical exertion for the patient. Consequently, PP imaging was performed with the patient’s torso leaning over the scanning device (minimal height of prone position imaging = 1 m). Correct head positioning and focus were achieved according to visual feedback provided by the infrared reflectance image (IR). Routine imaging in a sitting position was conducted using a regular SD-OCT device (Spectralis, Heidelberg Engineering^®^), which contained equivalent optical hardware and software. Both imaging methods were performed directly before the procedure (≤3 h), 3 h after, and at regular follow-up visits during the first 3 post-procedural weeks. If possible (depending on the patient’s cooperation and physical ability), 30° and 50° scans were obtained vertically and horizontally, respectively.

### 2.3. Morphological Review of SD-OCT Data

Prior to the procedure, SD-OCT scans were evaluated to determine whether there were any gravity-influenced configurational changes in the vitreomacular interface (VMI). In particular, the scans were examined for any changes in the configuration of retrohyaloidal fluid (RHF) and the PHM. No statistical analysis or area measurements were performed due to the limited sample size of this study. Subsequent clinical scans were comprehensively reviewed by two experienced clinicians to assess the morphological configuration, integrity, and reflectivity of the PHM, as well as its distinction from the posterior border of the gas bubble covering the VMI in a tilt-down position. Additionally, intraretinal integrity and position-dependent configurational changes in existing traction-related cysts were evaluated. PVD was diagnosed by SD-OCT and by funduscopic evidence. PVD was considered to be complete if both vitreous posterior adhesion (VPA) and VMT release were demonstrated in regular SD-OCT scans (in the sitting position). Given the exploratory nature of this study, the applied imaging method itself was evaluated for possible sources of error, morphological artefacts, and clinical significance.

### 2.4. Outcome Measures

Pre- and post-interventional IR images and SD-OCT scans were analyzed for (1) position-dependent changes in VMT and RHF configuration before intravitreal gas injection; (2) position-dependent changes of VMT and RHF configuration after intravitreal gas injection.

## 3. Results

**Figure 1:**
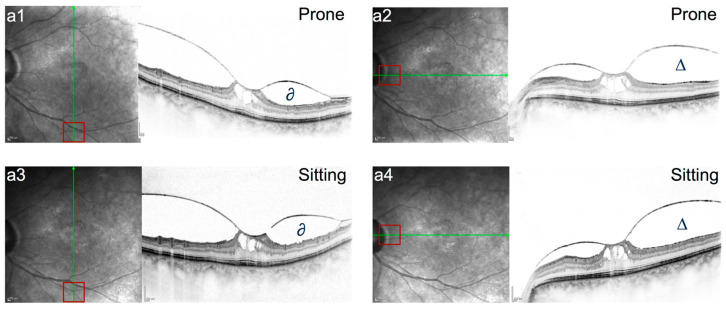
(**a1**–**a4**) Infrared reflectance (IR) and SD-OCT (30°) of a 70-year-old female patient (case a) with focal VMT, concomitant ERM, and broad vitreoretinal adhesion near the superior vascular arcades; images **a1**,**a2**: prone position; images **a3**,**a4**: sitting position; ∆ and ∂ mark the retrohyaloidal space, which shows no perceived differences in the prone and sitting positions. The red squares in the IR image highlight the limited possibility of exact/direct measurements between **a1**/**a2** and **a3**/**a4**.

**Figure 2:**
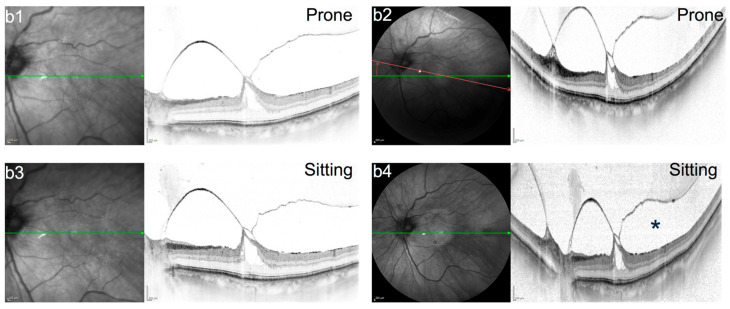
(**b1**–**b4**): IR and SD-OCT (30° + 50°) of a 79-year-old male patient with focal VMT, a large traction-related pseudocyst, concomitant ERM, and vitreopapillary adhesion showing advanced vitreous destruction; Images **b1**,**b2** obtained in prone position; Images **b3**,**b4** obtained in regular sitting position after obtaining **b1**,**b2**; * marks retrohyaloidal space (RHS); red line (**b2**) visualizes scan/eye rotation of **b4** compared to **b2** (12° clockwise).

**Figure 3:**
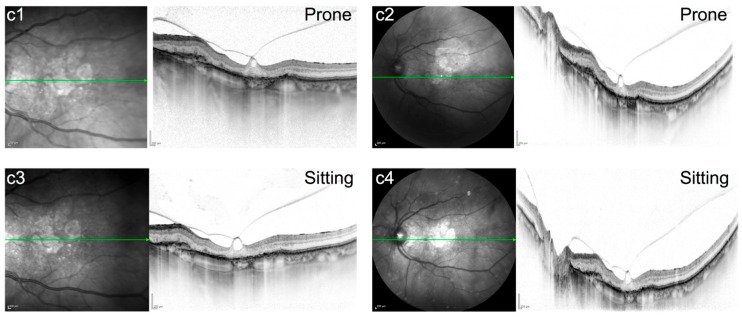
(**c1**–**c4**): IR and SD-OCT (30° + 50°)of an 82-year-old male patient with focal VMT, advanced dry AMD with geographic atrophy and persistent VPA. Images **c1**,**c2** were taken in prone and **c3**,**c4** in sitting position.

The data of three patients were analyzed. Patients’ clinical and morphological characteristics are described in Table 1 and in the captions of Figure 1, Figure 2 and Figure 3 (case a–c). Overall, VMT release after intravitreal gas injection was observed in case b and case c at six days and one day post-injection, respectively. Case a showed no PVD induction 3 weeks after the procedure, which led to the decision for pars plana vitrectomy three months after first treatment.

1. Positional Changes in the VMI configuration before intravitreal gas injection

Scans prior to pneumatic vitreolysis did not show any positional change in the RHF or PHM configuration between the sitting and prone position (Figure 1, Figure 2 and Figure 3). Figure 1, Figure 2 and Figure 3 show the baseline morphology of the VMI of all three patients (cases a–c) before the intravitreal gas injection.

2. Positional changes of the VMI configuration after intravitreal gas injection

**Figure 4:**
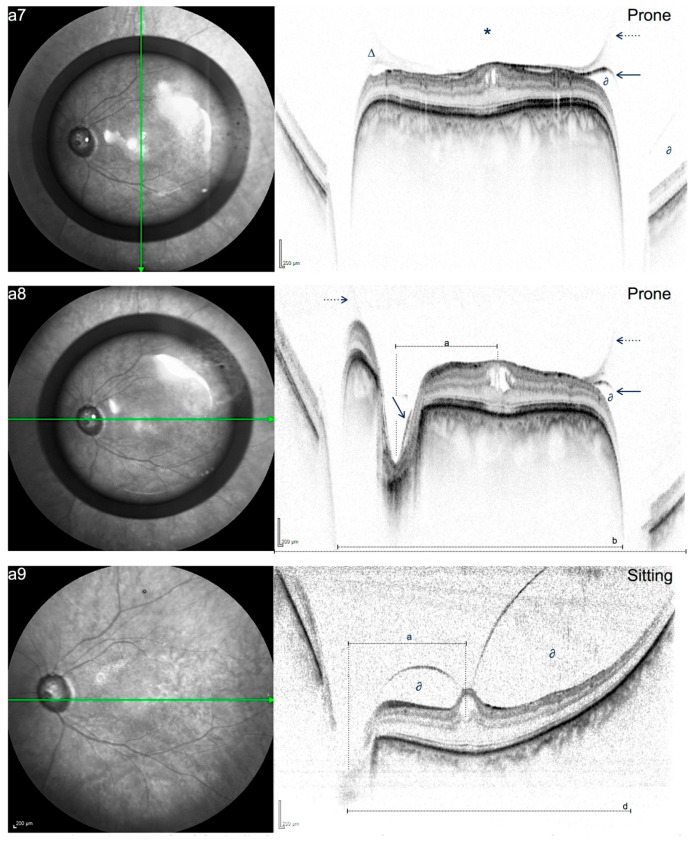
(**a7**–**a9**): IR and SD-OCT (50°) of case a, 15 days after surgery, showing the configuration change in VMT and PHM as the posterior pole is covered by the gas bubble in prone position (**a7**,**a8**); no VMT release can be documented. **a9** obtained immediately after **a7**/**a8**; regular arrow in **a7**,**a8** marks hyperreflective membrane correlating with the posterior hyaloid membrane (PHM). Dotted arrow distinguishes hyporeflective and more diffuse aspect of the posterior margin of the gas bubble; ∆ marks an area of moderate reflectivity likely correlating with condensed vitreous between the gas bubble and the PHM. * marks a hyporeflective area correlating with the gas bubble. In **a8** and **a9**, the retrohyaloid space is suspended by the gas bubble. The retrohyaloidal fluid seems to be shifted to the (mid)peripheral areas (marked with ∂ in both images); note that the PHM rotates in the same direction (downward) as the retinal surface, in contrast to the posterior contour of the gas bubble (upward). Due to the demagnifying effect of the bubble, the retinal area covered by the gas bubble correlates to an image angle of 41.2°, whereas a B-scan segment of the same length would correlate to a captured angle of approximately 35.4°. The bubble in this case was estimated to have a demagnifying effect of ~15%. (a = 14.8°; b = 41.2°; c = 50°; d = 53.4°).

**Figure 5:**
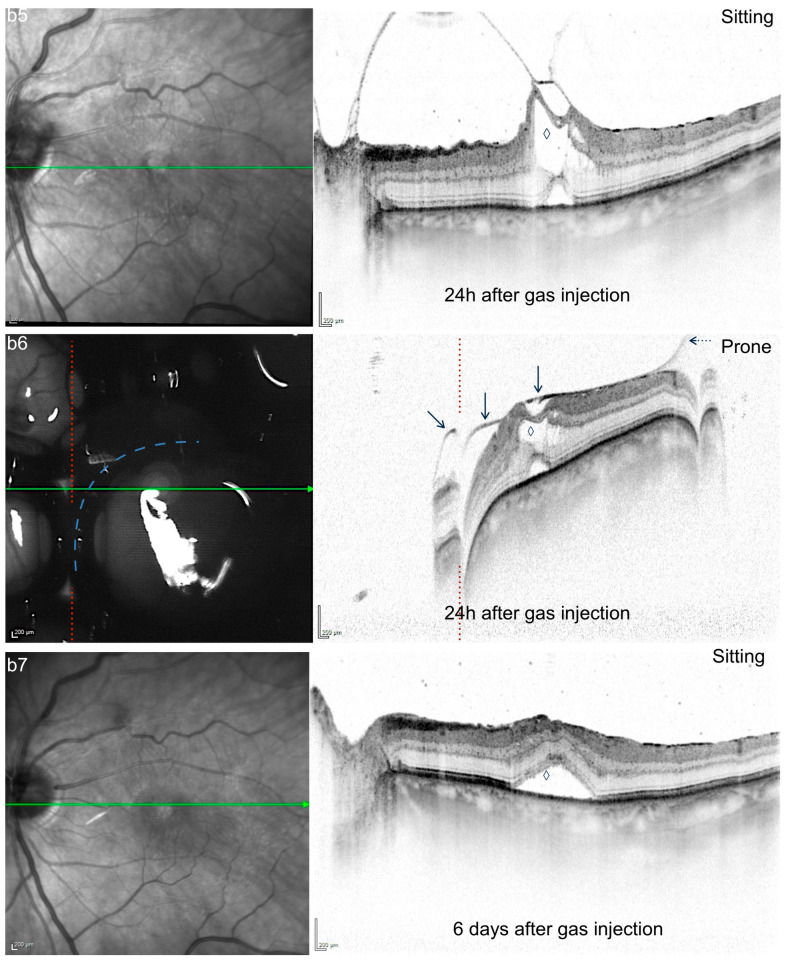
(**b5**–**b7**): IR and SD-OCT (30°) of case b showing configuration change in VMT and PHM one day after surgery (**b5**,**b6**) and VMT release 6 days after surgery (**b7**). **b5** was taken in regular sitting position immediately before **b6**; regular arrow in **b6** marks the PHM bridging the altered foveal contour, which can be clearly distinguished from the hyporeflective and more diffuse aspect of the posterior edge of the gas bubble (dotted arrow); ◊ marks a traction-related cyst in **b5**/**b6** and persistent subretinal fluid in **b7**. Red-dotted line marks “disruption artifacts” by the overlying gas bubble correlating to the nasal border of the central gas bubble (blue-dotted line).

**Figure 6:**
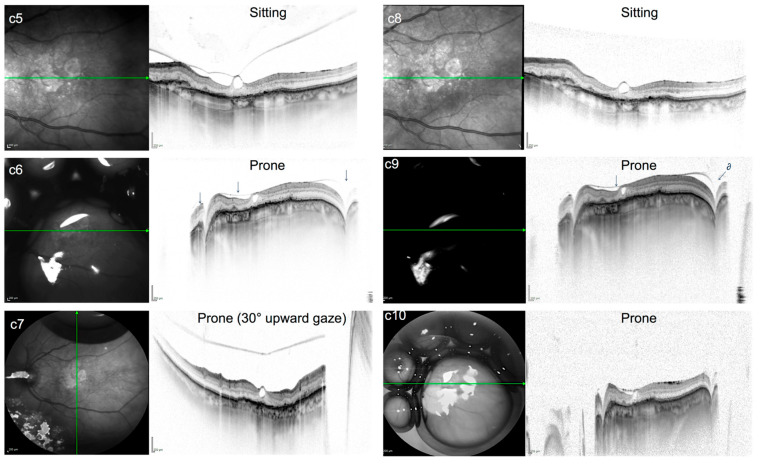
(**c5**–**c10**): IR and SD-OCT (30° + 50°) of case c before (**c5**,**c6**), during (**c6**) and after PVD induction. Images **c5**–**c7** were obtained 3 h after the injection of gas. **c8**–**c10** were obtained 24 h after the injection of gas. IR image of **c5**–**c7** shows a paw-like bubble configuration covering the macula; regular arrow marks the posterior hyaloid membrane (PHM). **c7** was obtained while the patient was instructed to follow the clinician’s fingertip in the face-down position, directing slow radial movements of 20–30° scale leading to spontaneous PHM detachment; 21 h later (**c8**), no PHM can be detected in the sitting position. In contrast, in the prone position, the intact PHM can be visualized as it is being pressed to the retinal surface by the intravitreal gas bubble (**c9**,**c10**). Typical morphologic features of the PHM, including high reflectivity, as well as a downward trend corresponding to the trend of all retinal layers, can be seen (regular arrow with ∂), demonstrating that vitreofoveolar traction has been released without the bubble breaking or crossing the PHM.

In post-procedural SD-OCT examinations in PP, the posterior contour of the gas bubble was located within the vitreous, more specifically anterior to the hyperreflective line correlating with the PHM. Figure 4, Figure 5 and Figure 6 show post-procedural scans taken in the sitting and prone positions. Table 1 describes the individual time points of examination and VMT/VPA release, if documented. In case c, immediate PVD induction was observed while images were obtained in the prone position. The detailed image sequence is therefore part of Figure 3.

In case c (Figure 6), immediate PVD induction was observed 3 h after the procedure, while the patient was instructed to make small direct eye movements in the face-down position that would allow the bubble to glide over the posterior pole moving radially back and forth between the fovea and the vascular arcade. In this case, the PHM could not be detected in the sitting position, while it was seen to be pressed against the retinal surface in the prone position one day after the procedure, with the gas bubble still located anterior to the PHM.

## 4. Discussion

Pneumatic vitreolysis describes the induction of posterior vitreous detachment by introducing a gas bubble into the vitreous cavity in patients with symptomatic vitreomacular traction. So far, no study is known to the authors that has visualized the mechanisms behind pneumatically induced PVD using OCT. In the present study, we were able to show the morphologic interaction of intravitreal gas with the adjacent vitreomacular interface using SD-OCT imaging in the prone position.

Our study found that the posterior edge of the intravitreal gas bubble previously injected into the vitreous gel could be successfully visualized pressing the PHM against the retinal surface in the prone position, resulting in a significant change in the configuration of the VMI and thus resulting in a temporary change of the tractional vectors.

Historically, the histomorphology and mechanics of intravitreal gas have been the focus of extensive experimental studies since its introduction into retinal detachment surgery by Lincoff in 1967 and later by Norton in 1973 [16]. At that time, a number of studies, mostly performed in rabbit eyes, focused on the morphologic consequences of intravitreal gas expansion on the posterior hyaloid surface, depicting displacement and compression of the vitreous gel, as well as observed PVD induction using transmission and scanning electron microscopy and biochemical assays [17,18,19]. In 1984, Miller et al. described PVD induction in rabbit eyes after the instillation of 0.4 mL of C3F8 in between 2 and 8 weeks [17]. The injected intravitreal gas was shown to completely fill the vitreous cavity after 3 days, leaving the central vitreous “empty”, while condensed vitreous was found at the margins aligning with the retinal surface. As the bubble gradually shrank, the condensed vitreous was described to regain water and some of its original structure, leading to vitreoretinal separation [17]. These findings were consistent with other morphologic studies of intravitreal gas, while Faulborn and colleagues in 1987 suggested that 6 months after vitreous displacement by intravitreal gas, the vitreous structure could in fact appear completely physiologic, with no evidence of condensation remaining [18,20]. Lincoff et al. reported significant disorganization of the collagen structure, which may destabilize the vitreous after gas absorption [19]. Based on these findings, it was proposed and elaborated by Chan et al. in 1995 that primary gas-mediated liquefaction (synchisis) with subsequent regeneration and swelling of the compressed vitreous after bubble absorption could lead to rupture of the posterior hyaloid cortex, resulting in the induction of PVD by fluid entering the retrohyaloid space from the collapsed (syneretic) cavity [12].

In terms of pneumatic vitreolysis, the current data describes successful release most frequently at a mean time ranging from about 2 to 4 weeks, a time frame coherent with the assumption that intravitreal gas would interfere with forementioned interplay of syneresis and synchesis [12]. However, Mori et al. pointed out that successful PVD would rather occur “during the days immediately after gas injection (…) rather than late (in weeks 2–4 of intraocular gas treatment)” [14]. In their retrospective review of 20 eyes treated with PV, Claus et al. revealed successful VMT release in 11 eyes during the first month [21]. However, in seven of these cases, VMT release was documented 14 days after injection, which for these patients was also the date of first presentation following the procedure. One patient was shown to have had immediate PVD induction (on the same day) [12]. Thus, it may be assumed that actual PVD induction might happen earlier than detected and described in the current literature, possibly hinting at other additive mechanisms leading to successful PVD induction. Could gas-mediated PVD—in contrast to physiological PVD—rather constitute an acute event, possibly dynamically influenced by certain eye movements—an idea originally manifested by Johnson et al. [2]? Given our imaging results, it seems likely that early gas-mediated PVD induction could occur resulting from a dynamic interplay of mechanical stress on the posterior hyaloid membrane and the sudden displacement of retrohyaloidal fluid.

**Figure 7:**
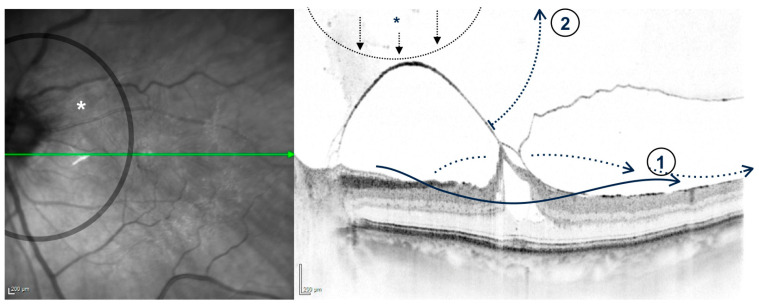
Schematic visualization of positioning a gas bubble (*) to the optic nerve head (IR image) with consecutive (1) retrohyaloidal fluid shift and (2) focal stress on the posterior hyaloid membrane, possibly resulting in increased anteriorly directed vectors of force inserted at the perifoveal traction site.

Based on this reasoning, we hypothesize that directing the gas bubble toward the optic nerve head, while leaving the fovea uncovered, could generate anteriorly directed force vectors concentrated on the perifoveal region (Figure 7).

Future prospective interventional studies may explore the use of smaller intravitreal gas bubbles to reduce the risk of retinal tears, while leveraging targeted posturing strategies to direct the gas bubble toward parafoveal regions according to a standardized positioning protocol.

The limitations of this study include its exploratory nature, retrospective design, and, most importantly, the absence of clinical endpoints due to the very small sample size of only three patients. As this non-interventional retrospective study was primarily conducted to assess the feasibility of prone position imaging and to describe the gas–retina interaction on OCT, no conclusions could be drawn regarding posturing regimens or complications such as retinal detachment.

## 5. Conclusions

Using prone position imaging, this study demonstrated that it is feasible to monitor and analyze the behavior of an injected intravitreal gas bubble with respect to its intraocular location, configuration (e.g., single versus multiple bubbles), and morphological impact as it covers and interacts with the posterior pole.

We hypothesize that, in addition to structural and biochemical alterations of the vitreous, early gas-mediated induction of posterior vitreous detachment may be driven by the interplay of two principal mechanisms. First, displacement of retrohyaloidal fluid may lead to the progressive release of midperipheral vitreoretinal adhesions. Second, focal amplification of perifoveal force vectors may occur as a result of mechanical stress on the posterior hyaloid membrane.

Together, the dynamic redistribution of retrohyaloidal fluid and the mechanical disruption of an otherwise rigid, self-sustaining system of vectorial forces with repeated and increasingly focused stress on the posterior hyaloid membrane may ultimately precipitate, and perhaps more abruptly than commonly assumed, the release of vitreomacular traction.

Potential clinical implications, including the role of specific posturing maneuvers, therefore warrant further systematic investigation, particularly in light of the reported risks associated with pneumatic vitreolysis. Addressing these issues will likely raise new questions that may be explored in greater detail through more frequent, comprehensive, and, most importantly, prospective imaging studies, offering insights from “behind the bubble.”

## Figures and Tables

**Table 1:** Clinical and morphological course of patients treated with pneumatic vitreolysis.

	Preoperative						Postoperative				
Patient	Age/Gender	Lens Status	BCVA (logMAR)	Amsler Grid	VMT Size (in µm)	CMT (in µm)	VMT Release (Time After PV)	VPA Release (Time After PV)	VMT Size	CMT (in µm) *	BCVA (logMAR) *
Case a	79/f	phakic	0.2	−	890	391	no	no	stable	231	0.1
Case b	70/m	IOL	0.3	+	463	819	yes (6 days)	yes (6 days)	N/A	432	0.5
Case c	82/m	IOL	0.4	+	142	265	yes (3 h)	yes (21 h)	N/A	231	0.3

Abbreviations: BCVA = Best Corrected Visual Acuity; * at last available follow-up; Case a: 5 months after intervention, 2 months after combined phacovitrectomy. Case b: 3 weeks; Case c: 1 week; VMT = Vitreomacular Traction; VPA = Vitreopapillary adhesion; CMT = Central Macular Thickness, measured in the center of the fovea perpendicularly to the RPE; f = female; m = male; IOL = intraocular lens.

## Data Availability

Data is available on request. Please contact the corresponding author.
